# Heterologous Expression and Purification Systems for Structural Proteomics of Mammalian Membrane Proteins

**DOI:** 10.1002/cfg.218

**Published:** 2002-12

**Authors:** Isabelle Mus-Veteau

**Affiliations:** Laboratoire de Physiologie Cellulaire et Moléculaire UMR-CNRS 6548 Université de Nice-Sophia Antipolis Parc Valrose Nice cedex 2 06108 France

## Abstract

Membrane proteins (MPs) are responsible for the interface between the exterior and the interior of the cell. These proteins are implicated in numerous diseases, such as cancer, cystic fibrosis, epilepsy, hyperinsulinism, heart failure, hypertension and Alzheimer's disease. However, studies on these disorders are hampered by a lack of structural information about the proteins involved. Structural analysis requires large quantities of pure and active proteins. The majority of medically and pharmaceutically relevant MPs are present in tissues at very low concentration, which makes heterologous expression in large-scale production-adapted cells a prerequisite for structural studies. Obtaining mammalian MP structural data depends on the development of methods that allow the production of large quantities of MPs. This review focuses on the different heterologous expression systems, and the purification strategies, used to produce large amounts of pure mammalian MPs for structural proteomics.


**Comparative and Functional Genomics**
*Comp Funct Genom* 2002; **3**: 511–517.
Published online in Wiley InterScience (www.interscience.wiley.com). **DOI:** 10.1002/cfg.218

**Conference Review**

### Heterologous expression and purification systems for structural proteomics of mammalian membrane proteins

Isabelle Mus-Veteau*
*Laboratoire de Physiologie Cellulaire et Moléculaire, UMR-CNRS 6548, Université de Nice-Sophia Antipolis, Parc Valrose, 06108 Nice cedex 2, France*

**Correspondence to:
Isabelle Mus-Veteau, Laboratoire de Physiologie Cellulaire et Moléculaire, UMR-CNRS 6548, Université de Nice-Sophia Antipolis, Parc Valrose, 06108 Nice cedex 2, France.
E-mail:
Isabelle.Mus-Veteau@unice.fr*

Received: 9 August 2002
Accepted: 14 October 2002

### Abstract

**Membrane proteins (MPs) are responsible for the interface between the exterior and the interior of the cell. These proteins are implicated in numerous diseases, such as cancer, cystic fibrosis, epilepsy, hyperinsulinism, heart failure, hypertension and Alzheimer's disease. However, studies on these disorders are hampered by a lack of structural information about the proteins involved. Structural analysis requires large quantities of pure and active proteins. The majority of medically and pharmaceutically relevant MPs are present in tissues at very low concentration, which makes heterologous expression in large-scale production-adapted cells a prerequisite for structural studies. Obtaining mammalian MP structural data depends on the development of methods that allow the production of large quantities of MPs. This review focuses on the different heterologous expression systems, and the purification strategies, used to produce large amounts of pure mammalian MPs for structural proteomics. Copyright © 2002 John Wiley & Sons, Ltd.**

**Keywords:** mammalian membrane protein; heterologous expression systems; purification strategy; 3D structure; drug target

### Introduction

Proteomics is more than the systematic partitioning and referencing of all the proteins produced in an organism, it is also the study of how proteins change structure, interact with other proteins, and ultimately give rise to disease or health in an organism [1]. Integral membrane proteins (MPs) account for 20–25% of all open reading frames in fully sequenced genomes. These proteins are of central importance to living cells. They are required for transport processes, sensing changes in the cellular environment, transmission of signals, and control of cell–cell contacts. MPs are implicated in cancer, cystic fibrosis, epilepsy, hyperinsulinism, heart failure, hypertension and Alzheimer's disease, but studies on these and other disorders are hampered by a lack of information about the proteins involved. Knowing the structure of MPs is an essential prerequisite to understanding how MPs function and, further, how their functions can be modified by small molecules. This is of paramount importance in the pharmaceutical industry, which produces many drugs that bind to MPs (e.g. Prozac and Imigram), and recognizes the potential of many recently identified G protein-coupled receptors, ion channels and transporters as targets for future drugs. However, whereas high-resolution structures are available for a myriad of soluble proteins, three-dimensional (3D) structures have so far been obtained for only 34 intregral MPs, the majority of which are from prokaryotic organisms, with only five being mammalian MPs [2,3,4,5,6] (see **http://www.mpibp-frankfurt.mpg.de/michel/public/memprotstruct.html**). One of the major factors in dictating why these particular MPs were crystallized was their

Copyright © 2002 John Wiley & Sons, Ltd.

natural abundance, circumventing all the difficulties associated with overexpression. However, the majority of medically and pharmaceutically relevant MPs are present in tissues at very low concentration, making overexpression in heterologous cells suitable for large-scale production a prerequisite for structural studies. The relative ease of overexpression of many bacterial transporters is making them prime candidates for structure determination [7,8,9]. Mammalian MPs are far more difficult to purify in large amounts than prokaryotic MPs, due to the need to express them in heterologous systems to achieve large-scale production. This review focuses on the different heterologous expression systems and purification strategies used to produce amounts of pure mammalian MPs that are adequate for structural analysis.

### Heterologous expression systems

### Chinese hamster ovary (CHO) cells

For large-scale commercial production of therapeutically important proteins like erythropoietin, recombinant CHO (rCHO) cells have been adapted to grow in suspension in serum free media which is an advantage in terms of cost, as well as of biosafety [10]. This suspension culture of rCHO cells has been shown to be an efficient system for expressing the rat 5-$HT_2$ serotonin receptor for drug discovery [11], but no MP has been purified from this host in an amount sufficient for structural analysis.

### Insect cells

Baculovirus-mediated expression in insect cells such as *Spodoptera frugiperda* (SF9) is a well-established approach for the production of recombinant glycoproteins. Like rCHO cells, insect cells can be grown in suspension in serum free media. The rat synaptic vesicle monoamine transporter [12] and the human $\alpha 1$ glycine receptor [13] have been functionally overexpressed and purified from baculovirus-mediated expression systems, yielding 0.5 mg and 1 mg pure protein/l cell culture, respectively (Table 1). Recently, heterologous genes have been shown to be expressed by stable transfection of insect cells [14]. The major advantage of this host for expression of mammalian MPs is that many processing events known in mammalian systems also occur in insects [15]. However, large-scale production of MPs using this system is time-consuming and expensive.

### Bacteria

Prokaryotic homologues have been most amenable for obtaining structural information on MPs because they can often be expressed in bacteria in large quantities [16]. However, this method cannot easily be extended to mammalian MPs, since these proteins are mostly expressed in inclusion bodies, from which they are usually impossible to purify under non-denaturing conditions. However, derivatives of *E. coli* BL21(DE3) strain, CD41(DE3) and CD43(DE3), selected to grow to high saturation cell density and to overproduce proteins without toxic effect for the host cell [17], have been successfully used for production of mitochondrial MPs [18,19]. Very recently, the human $Na^+$/glucose transporter has been functionally expressed and purified using an *E. coli* BL21(DE3) mutant (defective in the outer membrane protease OmpT) and incubation temperatures below 20 °C, reducing proteolytic degradation (Table 1) [20]. The green fluorescent protein (GFP) reporter was used to show that this $Na^+$/glucose recombinant transporter was inserted into the bacterial plasma membrane and not into inclusion bodies [21,20]. In contrast to the case for the serotonin transporter, which could not be functionally expressed in *E. coli* [22], cholesterol (a steroid present in eukaryotic membranes but not in *E. coli*) and N-glycosylation (bacteria do not possess glycosylation machinery) are not required for the glucose transporter to show activity.

### Yeast

This simple eukaryotic cell is a multi-purpose host, performing many of the post-translational modifications seen in higher eukaryote cells (glycosylation, disulphide bond formation and proteolytic processing), combined with the ease of growing a large volume of cells in short period of time. Cloning, functional expression, identification of interacting partners, mutagenesis of the protein, or its partners, and overexpression for biophysical analysis can all be achieved in yeast [23]. Exciting possibilities also exist for the exploitation of yeast genetics to tailor yeast strains

Copyright © 2002 John Wiley & Sons, Ltd. 

**Table 1.** Examples of mammalian MPs successfully expressed and purified in heterologous expression systems

| MPs expressed | Heterologous expression systems | Purification procedure | Amount of purified MP per culture (mg/l) |
|---|---|---|---|
| Rabbit SERCA1a $Ca^{2+}$-ATPase [26] | Yeast *S. cerevisiae* | $Ni^{2+}$ affinity chromatography followed by reactive red chromatography | 0.05 |
| Rat vesicular monoamine transporter [24] | Yeast *S. cerevisiae* | $Ni^{2+}$ affinity chromatography | nd |
| Human P-glycoprotein [25] | Yeast *S. cerevisiae* | $Ni^{2+}$ affinity chromatography | 0.4 |
| N-glycosylation mutant mouse and human P-glycoproteins [31] | Yeast *P. pastoris* | $Ni^{2+}$ affinity chromatography | 1.0 |
| Mutant mouse P-glycoprotein [30] | Yeast *P. pastoris* | $Ni^{2+}$ affinity chromatography | 1.0 |
| Biotinylated mouse P-glycoprotein [29] | Yeast *P. pastoris* | $Ni^{2+}$ and avidin affinity chromatography | 1.4 |
| Human peptide transporter [32] | Yeast *P. pastoris* | $Ni^{2+}$ affinity chromatography | 0.2 |
| Human $Na^{+}$/glucose transporter [21] | Bacteria *E. coli*, BL21(DE3) | Anti-FlagM2 affinity chromatography | 0.3 |
| Human $\alpha$1 glycine receptor [13] | Insect cells | Aminostrychnine affinity chromatography | 1.0 |
| Rat synaptic-vesicle monoamine transporter [12] | Insect cells | $Ni^{2+}$ affinity chromatography | 0.5 |

nd, not determined.

so that they are optimized for the expression of MPs. Two yeast species have so far been used successfully for heterologous expression of mammalian MPs: *Saccharomyces cerevisiae* and *Pichia pastoris*.

*Saccharomyces cerevisiae* has been successfully used to functionally express and purify several mammalian MPs, like the human P-Glycoprotein [24], the rat vesicular monoamine transporter [25] and, very recently, the rabbit SERCA1a $Ca^{2+}$-ATPase [26] (Table 1). The expression vectors used are 2μ multicopy plasmids with the inducible *GAL1* promoter or the strong constitutive promoter from the yeast plasma membrane ATPase (*PMA1*). Fliger *et al.* [24] show that human P-glycoprotein expression can reach 8% of total MPs using a protease-deficient strain, and glycerol (thought to enhance the post-translational stability of the protein). The level of expression reached is comparable to that of mammalian cells (COS, HEK293), yet at only a fraction of the cost, time and effort. Yelin and Schuldiner [25] showed that the expression of the vesicular monoamine transporter was enhanced by modification of certain codons in the translation initiation region to codons preferred by *S. cerevisiae*, and by reducing the temperature to 16 °C, which also allowed the expressed transporters to undergo core glycosylation. Lowering the temperature from 30 °C to 16–18 °C seems to be a general rule for enhancing the functional expression of MPs, since it has also been observed for the SERCA1a $Ca^{2+}$-ATPase [26].

The methylotrophic yeast, *P. pastoris*, has been used for the expression of more than 300 heterologous proteins and has been used successfully as a tool for large-scale recombinant soluble protein production [27]. *P. pastoris* is a poor fermenter, a major advantage relative to *S. cerevisiae*. In high cell density cultures, ethanol (a product of *S. cerevisiae* fermentation) rapidly builds to toxic levels that limit further growth and foreign protein production. With its preference for respiratory growth, *P. pastoris* can be cultured at extremely high densities ($500_{OD600}$ U/ml) in the controlled environment of a fermenter. Moreover, foreign genes are stably integrated in single or multiple copy behind the *AOX1* (alcohol oxidase 1) promoter, one of the strongest, most regulated promoters known. However, the full potential for *P. pastoris* as a host for MPs is yet to be realized, although promising results have been obtained with the purification of about 0.2 mg/l culture of the human $\beta$2 adrenergic receptor [28], the human and mouse P-glycoproteins [29,30,31] and the human peptide transporter [32] (Table 1).

Although yeast appears to be the most amenable expression system for many mammalian MPs, some MPs cannot be functionally expressed in yeast, e.g. the serotonin transporter (SERT) can only be functionally expressed in insect and

Copyright © 2002 John Wiley & Sons, Ltd. 

mammalian cells due to the requirement for: N-linked glycosylation (for folding and stability of the transporter), the molecular chaperone calnexin (involved in the folding of SERT) and cholesterol (to maintain the protein structure) [22].

### Purification strategies

Most of the mammalian MPs successfully expressed in heterologous systems have been purified using a single-step affinity chromatography $Ni^{2+}$-NTA column that binds a polyHis tag fused at the N- or C-terminus of the protein (Table 1). This single purification step results in a 70–80% enrichment of the protein of interest. Further chromatographic steps are required to obtain pure protein, often resulting in a dramatic reduction in the amount of protein.

Séraphin and collaborators have developed a purification strategy allowing efficient recovery of proteins present at low levels in the cell: the tandem affinity purification (TAP) method [33]. IgG-binding units of protein A from *Staphylococcus aureus* (ProtA) and the calmodulin binding peptide (CBP) were fused in tandem, linked by a TEV cleavage site. The TAP tag is fused to the protein of interest and the construct is introduced into the cognate host cell or organism. The TAP method has been optimized for rapid purification of proteins expressed at their natural level, under native conditions, allowing protein complex identification and proteome exploration [34]. This method could be useful for large-scale purification of mammalian MPs. The fusion of a TAP tag at the N- or C-terminus of the MP of interest would allow the use of two affinity chromatography steps for efficient recovery of that MP. Because it is generic and rapid, the TAP procedure may constitute an important new tool for structural proteomics of MPs.

### Structural analysis

Structural information is a powerful tool for understanding the working mechanisms of macromolecules at the molecular level. Structural proteomics is therefore of great interest to the pharmaceutical industry: the 3D structure of biomedical target proteins can be exploited to develop small molecule effectors against diseases. At the fundamental level, structural proteomics is a cornerstone of functional genomics: the uncovering of the biochemical and cellular function of all ORFs in an organism. The 3D structure of a protein can be used to deduce function from unknown proteins, to make molecular models of related proteins, which can then serve biochemical studies and to predict interactions with small molecule effectors or protein partners (van Tilbeurgh, 'From Genome to Life' Cargèse Summer School 2002). The genomic approach that consists of resolving the structure of several bacterial members of an MP family has been developed for different types of MPs, such as the ABC transporters MsbA and BtuCD [9,35] and chloride channels [8]. Other groups have chosen to perform crystallographic studies on MPs from thermophilic archaea because they are more stable and easier to purify [36,37]. This approach is an alternative to mammalian MP structure determination because bacterial MPs are usually easier to express, purify and crystallize, and the 3D structures can serve as models for the study of their mammalian homologues.

The different structural proteomics projects under development for *Methanobacterium thermoautotrophicum* [36] and yeast (**http://genomics.eu.org/**) provide databases with a number of soluble protein structures, but very few MP structures.

For X-ray crystallography, the techniques used are nearly identical to that used for soluble proteins, except that the object being crystallized is not just protein but a protein–detergent complex. Obtaining crystals that diffract well requires a homogeneous protein–detergent complex that may be affected by the presence of impurities, especially lipids, which are difficult to remove because of their stabilizing effect. Despite these difficulties, the number of 3D structures of integral MPs is increasing rapidly: 21 of the 34 known integral MP structures have been solved in the last 3 years (see **http://www.mpibp-frankfurt.mpg.de/michel/public/memprotstruct.html**).

Two methods have improved the success rate in 3D crystallization: the use of antibody Fv fragments to increase the effective hydrophilic area of the MP [38,39,40]; and 3D crystallization in cubic lipid phases, which provides an environment that allows solubilized MPs to diffuse and merge into micro-crystals within the labyrinth of the cubic lipid system [41,42,43,44].

Copyright © 2002 John Wiley & Sons, Ltd.

A powerful alternative is reconstitution into two-dimensional (2D) crystals in the presence of lipids [45], restoring the native environment of MPs as well as their biological activity. These crystals can be studied both by cryo-electron microscopy, which allows the 3D structure of the vitrified protein to be assessed at atomic resolution, and atomic force microscopy (AFM), which depicts biological membranes in aqueous solutions and permits the monitoring of the movement of single polypeptide loops. The combined application of these two techniques can establish the 3D structure of MPs and allow the visualization of their conformational changes during catalytic cycles [46].

Spectroscopic techniques such as fluorescence and infrared are useful to probe aspects of transmembrane segment orientation, structure and dynamics in lipid bilayers. The fluorescence from tryptophan contains valuable information about the local environment of the indole side-chain. This environment sensitivity, coupled with the ability to incorporate a single tryptophan residue at specific sites in a polypeptide sequence, has provided the membrane biophysicist with tools for examining the structure and dynamics of MPs [47,48].

### Conclusion

MPs represent one of the most challenging classes of proteins in the areas of overexpression/purification and structural biochemistry. The heterologous expression problems associated in particular with mammalian MPs are a major bottleneck to overcome in studies of the structure and function of these MPs. Purification of mammalian MPs such as the serotonin transporter (SERT) requires the growth of tens of litres of insect or mammalian cells, which is extremely time consuming and costly. The use of yeast, and to a lesser extent of bacteria, as hosts for mammalian MP overexpression has been heavily studied over the last few years and is becoming more and more successful. Some prerequisites seem to favour mammalian MP expression in these hosts: the modification of the codon usage of the initiation region with codons shown to stabilize the mRNA in yeast, the combined use of protease-deficient strains and a growth temperature of 16–18 °C to avoid proteolytic degradation and favour core glycosylation, and the use of chemical chaperones such as glycerol to stabilize proteins. Moreover, the tandem affinity purification strategy should allow more efficient purification and facilitate the production of pure MPs for structural studies. The recent progress in heterologous expression in bacteria and yeast, and in purification strategies, encourages the development of structural proteomics programs on MPs.

### Acknowledgements

I thank Dr Martine Bassilana (Institute of Signalling Development Biology and Cancer, Université de Nice-Sophia-Antipolis) for critical reading of this article.

### References

1. Brower V. 2001. Proteomics: biology in the post-genomic era. Companies all over the world rush to lead the way in the new post-genomics race. *EMBO Rep* **2**: 558–560.
2. Tsukihara T, Aoyama H, Yamashita E, *et al*. 1996. The whole structure of the 13-subunit oxidized cytochrome c oxidase at 2.8 Å. *Science* **272**: 1136–1144.
3. Iwata S, Lee JW, Okada K, *et al*. 1998. Complete structure of the 11-subunit bovine mitochondrial cytochrome *bc*1 complex. *Science* **281**: 64–71.
4. Palczewski K, Kumasaka T, Hori T, *et al*. 2000. Crystal structure of rhodopsin: a G protein-coupled receptor. *Science* **289**: 739–745.
5. Murata K, Mitsuoka K, Hirai T, *et al*. 2000. Structural determinants of water permeation through aquaporin-1. *Nature* **407**: 599–605.
6. Toyoshima C, Nakasako M, Nomura H, Ogawa H. 2000. Crystal structure of the calcium pump of sarcoplasmic reticulum at 2.6 Å resolution. *Nature* **405**: 647–655.
7. Legros C, Pollmann V, Knaus HG, *et al*. 2000. Generating a high affinity scorpion toxin receptor in KcsA-Kv1.3 chimeric potassium channels. *J Biol Chem* **275**: 16 918–16 924.
8. Dutzler R, Campbell EB, Cadene M, Chait BT, MacKinnon R. 2002. X-ray structure of a ClC chloride channel at 3.0 Å reveals the molecular basis of anion selectivity. *Nature* **415**: 287–294.
9. Locher KP, Lee AT, Rees DC. 2002. The *E. coli* BtuCD structure: a framework for ABC transporter architecture and mechanism. *Science* **296**: 1091–1098.
10. Lee GM, Kim EJ, Kim NS, Yoon SK, Ahn YH, Song JY. 1999. Development of a serum-free medium for the production of erythropoietin by suspension culture of recombinant Chinese hamster ovary cells using a statistical design. *J Biotechnol* **69**: 85–93.
11. Zaworski PG, Evans DL, Lahti RA, Gill GS. 1995. Growth of Chinese hamster ovary [CHO] cells expressing the 5-$HT_2$ serotonin receptor in suspension culture: an efficient method for large-scale acquisition of membrane protein for drug evaluation. *J Neurosci Methods* **56**: 169–175A.
12. Sievert MK, Thiriot DS, Edwards RH, Ruoho AE. 1998. High-efficiency expression and characterization of the

Copyright © 2002 John Wiley & Sons, Ltd. 

synaptic-vesicle monoamine transporter from baculovirus-infected insect cells. *Biochem J* **330**: 956–966.
13. Cascio M, Shenkel S, Grodzcki RL, Sigworth J, Fox RO. 2001. A Sm-like protein complex that participates in mRNA degradation. *J Biol Chem* **276**: 20 981–20 988.
14. Kempf J, Snook LA, Vonesch JL, Dahms TE, Pattus F, Massotte D. 2002. Expression of the human mu opioid receptor in a stable Sf9 cell line. *J Biotechnol* **95**: 181–187.
15. Altmann F, Staudacher E, Wilson IB, Marz L. 1999. Insect cells as hosts for the expression of recombinant glycoproteins. *Glycoconj J* **16**: 109–123.
16. Doyle DA, Cabral JM, Pfuetzner RA, *et al.* 1998. The structure of the potassium channel: molecular basis of $K^+$ conduction and selectivity. *Science* **280**: 69–77.
17. Miroux B, Walker JE. 1996. Overproduction of proteins in *Escherichia coli*: mutant hosts that allow synthesis of some membrane proteins and globular proteins at high levels. *J Mol Biol* **260**: 289–298.
18. Fiermonte G, Palmieri L, Dolce V, *et al.* 1998. The sequence, bacterial expression, and functional reconstitution of the rat mitochondrial dicarboxylate transporter cloned via distant homologs in yeast and *Caenorhabditis elegans*. *J Biol Chem* **273**: 24 754–24 759.
19. Fiermonte G, Palmieri L, Todisco S, Agrimi G, Palmieri F, Walker JE. 2002. Identification of the mitochondrial glutamate transporter. Bacterial expression, reconstitution, functional characterization, and tissue distribution of two human isoforms. *J Biol Chem* **277**: 19 289–19 294.
20. Drew DE, von Heijne G, Nordlund P, de Gier JW. 2001. Green fluorescent protein as an indicator to monitor membrane protein overexpression in *Escherichia coli*. *FEBS Lett* **507**: 220–224.
21. Quick M, Wright E. 2002. Employing *Escherichia coli* to functionally express, purify, and characterize a human transporter. *Proc Natl Acad Sci USA* **99**: 8597–8601.
22. Tate CG. 2001. Overexpression of mammalian integral membrane proteins for structural studies. *FEBS Lett* **504**: 94–98.
23. Bill RM. 2001. Yeast-a panacea for the structure–function analysis of membrane proteins? *Curr Genet* **40**: 157–171.
24. Figler RA, Omote H, Nakamoto RK, Al-Shawi MK. 2000. Use of chemical chaperones in the yeast *Saccharomyces cerevisiae* to enhance heterologous membrane protein expression: high-yield expression and purification of human P-glycoprotein. *Arch Biochem Biophys* **376**: 34–46.
25. Yelin R, Schuldiner S. 2001. Vesicular monoamine transporters heterologously expressed in the yeast *Saccharomyces cerevisiae* display high-affinity tetrabenazine binding. *Biochim Biophys Acta* **1510**: 426–441.
26. Lenoir G, Menguy T, Corre F, *et al.* 2002. Overproduction in yeast and rapid and efficient purification of the rabbit SERCA1a $Ca^{2+}$-ATPase. *Biochim Biophys Acta* **1560**: 67–83.
27. Cereghino JL, Cregg JM. 2000. Heterologous protein expression in the methylotrophic yeast *Pichia pastoris*. *FEMS Microbiol Rev* **24**: 45–66.
28. Weiss HM, Haase W, Michel H, Reiländer H. 1998. Comparative biochemical and pharmacological characterization of the mouse $5HT_{5A}$ receptor and the human $\beta$2-adrenergic receptor produced in the methylotrophic yeast *Pichia pastoris*. *Biochem J* **330**: 1137–1147.
29. Julien M, Kajiji S, Kaback RH, Gros P. 2000. Simple purification of highly active biotinylated P-glycoprotein: enantiomer-specific modulation of drug-stimulated ATPase activity. *Biochemistry* **39**: 75–85.
30. Lerner-Marmarosh N, Gimi K, Urbatsch IL, Gros P, Senior AE. 1999. Large scale purification of detergent-soluble P-glycoprotein from *Pichia pastoris* cells and characterization of nucleotide binding properties of wild-type, Walker A, and Walker B mutant proteins. *J Biol Chem* **274**: 34 711–34 718.
31. Urbatsch IL, Wilke-Mounts S, Gimi K, Senior AE. 2001. Purification and characterization of N-glycosylation mutant mouse and human P-glycoproteins expressed in *Pichia pastoris* cells. *Arch Biochem Biophys* **388**: 171–177.
32. Theis S, Döring F, Daniel H. 2001. Expression of the myc/His-tagged human peptide transporter hPEPT1 in yeast for protein purification and functional analysis. *Protein Expr Purif* **22**: 436–442.
33. Rigaut G, Shevchenko A, Rutz B, Wilm M, Mann M, Séraphin B. 1999. A generic protein purification method for protein complex characterization and proteome exploration. *Nature Biotechnol* **17**: 1030–1032.
34. Bouveret E, Rigaut G, Shevchenko A, Wilm M, Séraphin B. 2000. A Sm-like protein complex that participates in mRNA degradation. *EMBO J* **19**: 1661–1671.
35. Chang G, Roth CB. 2001. Structure of MsbA from *E. coli*: a homolog of the multidrug resistance ATP binding cassette (ABC) transporters. *Science* **293**: 1793–1800.
36. Morsomme P, Chami M, Marco S, *et al.* 2002. Characterization of a hyperthermophilic P-type ATPase from *Methanococcus jannaschii* expressed in yeast. *J Biol Chem* **277**: 29 608–29 616.
37. Christendat D, Yee A, Dharamsi A, *et al.* 2000. Structural proteomics: prospects for high throughput sample preparation. *Prog Biophys Mol Biol* **73**: 339–345.
38. Iwata S, Ostermeier C, Ludwig B, Michel H. 1995. Structure at 2.8 Å resolution of cytochrome c oxidase from *Paracoccus denitrificans*. *Nature* **376**: 660–669.
39. Hunte C. 2001. Insights from the structure of the yeast cytochrome *bc*1 complex: crystallization of membrane proteins with antibody fragments. *FEBS Lett* **504**: 126–132.
40. Zhou Y, Morais-Cabral JH, Kaufman A, MacKinnon R. 2001. Chemistry of ion coordination and hydration revealed by a $K^+$ channel–Fab complex at 2.0 Å resolution. *Nature* **414**: 43–48.
41. Nollert P, Qiu H, Caffrey M, Rosenbusch JP, Landau EM. 2001. Molecular mechanism for the crystallization of bacteriorhodopsin in lipidic cubic phases. *FEBS Lett* **504**: 179–186.
42. Pebay-Peyroula E, Rummel G, Rosenbusch JP, Landau EM. 1997. X-ray structure of bacteriorhodopsin at 2.5 Å from microcrystals grown in lipidic cubic phases. *Science* **277**: 1676–1681.
43. Kolbe M, Besir H, Essen LO, Oesterhelt D. 2000. Structure of the light-driven chloride pump halorhodopsin at 1.8 Å resolution. *Science* **288**: 1390–1396.
44. Luecke H, Schobert B, Lanyi JK, Spudich EN, Spudich JL. 2001. Crystal structure of sensory rhodopsin II at 2.4 Å: insights into color tuning and transducer interaction. *Science* **293**: 1499–1503.

Copyright © 2002 John Wiley & Sons, Ltd. 

45. Levy D, Chami M, Ridaud JL. 2001. Two-dimensional crystallization of membrane proteins: the lipid layer strategy. *FEBS Lett* **504**: 187–193.
46. Stahlberg H, Fotiadis D, Scheuring S, *et al*. 2001. Two-dimensional crystals: a powerful approach to assess structure, function and dynamics of membrane proteins. *FEBS Lett* **504**: 166–172.
47. Clayton AH, Sawyer WH. 2002. Site-specific tryptophan fluorescence spectroscopy as a probe of membrane peptide structure and dynamics. *Eur Biophys J* **31**: 9–13.
48. Cordat E, Leblanc G, Mus-Veteau I. 2000. Evidence for a role of helix IV in connecting cation- and sugar-binding sites of *Escherichia coli* melibiose permease. *Biochemistry* **39**: 4493–4499.

Copyright © 2002 John Wiley & Sons, Ltd. 
